# When too much of a *novel* thing may be what's “bad”: commentary on Fisher, Godwin, and Seltman (2014)

**DOI:** 10.3389/fpsyg.2014.01444

**Published:** 2014-12-23

**Authors:** Kana Imuta, Damian Scarf

**Affiliations:** ^1^School of Psychology, University of QueenslandBrisbane, QLD, Australia; ^2^Department of Psychology, University of OtagoDunedin, New Zealand

**Keywords:** classroom environment, attention, novelty response, children's education, children, learning and memory

Young children's classrooms are often filled with colorful decorations. In their recent article, Fisher et al. (2014a) present evidence that these decorations may be detrimental to children's learning. Specifically, children were less likely to stay focused, and attained lower test scores, when experimental lessons were given in a “decorated classroom” compared to a “sparse classroom.” Furthermore, children's test scores were negatively correlated with the amount of time that they were distracted, suggesting a direct relationship between these two variables. Fisher et al. (2014a) concluded that “colorful visual displays may promote off-task behavior in young children, resulting in reduced learning opportunities and achievement” (p. 1368). Is too much of a good thing bad? We argue here that, perhaps, only when the good things are all too novel.

Fisher et al. (2014a) suggest that extraneous visual stimuli compete for children's attention, causing distraction and impair performance on cognitive tasks. Rather than the *irrelevance* of the visual displays, however, it may have been the *novelty* that detracted children's attention away from the lesson to the environment. Indeed, Fisher et al.'s (2014a) decorated classroom was a laboratory room, purposefully adorned with a large amount of novel and colorful displays. Their sparse classroom, on the other hand, was the same room, but with all of these novel displays removed. It is well-established that novelty has powerful effects on children's attention allocation; in fact, some of the most well-established empirical procedures that are used to study early cognitive development rely on children's preference to attend to novel stimuli (Hayne, 2004). Fisher et al. (2014a) briefly allude to the issue of novelty in their Discussion, however, suggest that this cannot account for their findings.

Specifically, in their Discussion, Fisher et al. (2014a) cites another one of their studies in which children were given lessons in a decorated classroom for 2 weeks and, when compared to behavior in the sparse classroom, children were distracted for a greater amount of time in the decorated classroom on both Weeks 1 and 2 (Godwin and Fisher, 2012). Fisher et al. (2014a) did not acknowledge, however, that the proportion of time that children spent attending to the decorated environment decreased significantly between Weeks 1 and 2; that is, children exhibited habituation to the environment (Godwin and Fisher, 2012). If this is the case, classroom decorations may only have a transient impact on children's attention.

In their analysis, Fisher et al. (2014a) collapsed across the three lessons children spent in each classroom, potentially masking any habituation that may have occurred. To investigate this possibility, we reanalyzed their data (Fisher et al., 2014b). We used two methods to accommodate the fact that 8 (33%) children missed at least one of the six lessons: First, we conducted the ANOVA with list-wise deletion, such that only the 16 children with complete data sets were included. Second, we used the data imputation procedure in SPSS to replace missing data, such that all 24 children could be included in the analysis. Both methods resulted in identical pattern of results, so here we report only the outcomes from the former. Condition, *F*_(1, 15)_ = 53.16, *p* < 0.001, η^2^ = 0.78, and Lesson, *F*_(2, 30)_ = 5.59, *p* = 0.009, η^2^ = 0.27, were significant and qualified by a Condition × Lesson interaction, *F*_(2, 30)_ = 12.80, *p* < 0.001, η^2^ = 0.46. To investigate the interaction, separate One-Way ANOVAs were conducted for each condition with Lesson as a repeated measure. Lesson was significant for both the Sparse, *F*_(2, 30)_ = 8.07, *p* = 0.002, η^2^ = 0.35, and Decorated, *F*_(2, 30)_ = 9.18, *p* = 0.001, η^2^ = 0.38, conditions. As shown in Figure 1, the percentage of time that children were distracted by the environment increased slightly across lessons in the sparse classroom. More importantly, consistent with the idea that children may habituate to a decorated classroom, the percentage of time that children were distracted by the environment decreased considerably across lessons in the decorated classroom.

**Figure 1:**
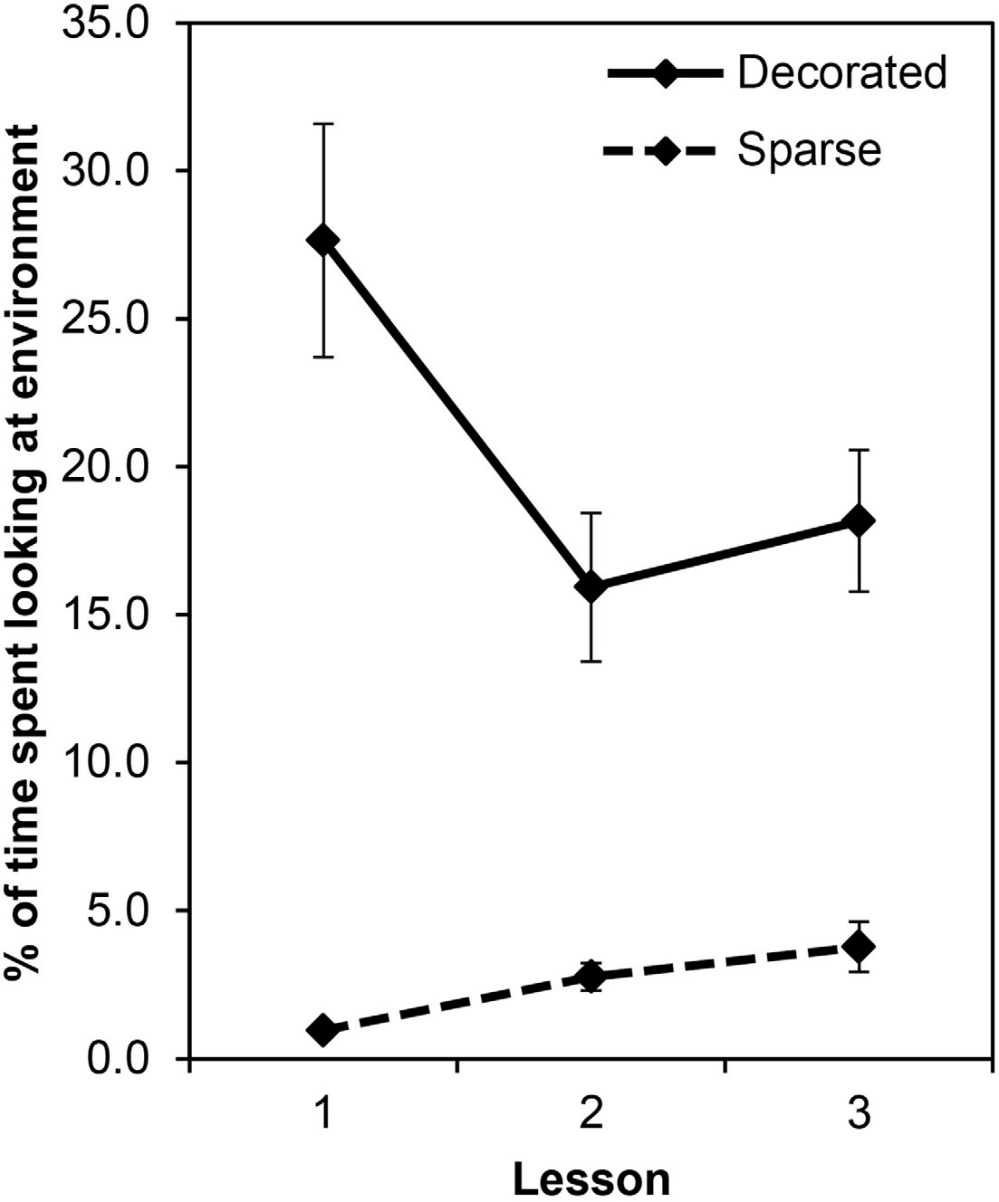
**Percentage of time that Fisher et al.'s (2014b) children spent looking at the classroom environment during their first, second, and third lessons in the “decorated” and “sparse” classrooms**.

On one hand, the lack of habituation between Lessons 2 and 3 in the decorated classroom suggests that, even with greater exposure, no further habituation will occur; however, we think this is unlikely for two reasons. First, children only spent approximately three 15-min sessions in each classroom, dispersed across a 2-week period. To put this into context, the 45 min children spent in each classroom represents, at most, just 25% of a half day (~3 h) and 13% of a full day (~6 h) of kindergarten, or 5 and 3% of a 5-day kindergarten week, respectively. Given this, the level of distraction displayed by children in Lessons 2 and 3 is likely not reflective of what one would observe in an actual kindergarten setting. Second, in another one of Fisher and colleagues' previous studies that assessed children's on- and off-task behaviors in actual elementary school classrooms, they reported that children spent just 4.6% of their time attending to the environment (Godwin et al., 2013). This level of distraction is essentially identical to that displayed by children in the sparse classroom in Lesson 3 (*M* = 4.7%), and supports our speculation that further habituation would have occurred, had the children in Fisher et al.'s (2014a) study spent more time in the decorated classroom. Keeping this in mind, however, we also acknowledge that at least within the scope of Fisher et al.'s (2014b) data, condition differences remained at Lesson 3. We propose, therefore, that our novelty hypothesis and Fisher et al.'s (2014a) irrelevance hypothesis may not be mutually exclusive.

Beyond a momentary distraction, classroom decorations may actually be beneficial in the long run. For example, allowing children to decorate the classroom with their own work may improve their self-esteem and beliefs about the value of their work (Maxwell and Chmielewski, 2008). Also, when classroom decorations are related to what the children are learning, the decorations may act as “reminder” cues, improving children's long-term memory of educational information (Hayne, 2004). Taken together, when considering the valuable question of how to optimize the classroom visual environment, we should consider how the relevance as well as the novelty of the visual displays interact to influence children's learning outcomes.

## Conflict of interest statement

The authors declare that the research was conducted in the absence of any commercial or financial relationships that could be construed as a potential conflict of interest.
